# Multiparametric Magnetic Resonance Imaging for Immediate Target Hit Assessment of CD13—Targeted Tissue Factor tTF-NGR in Advanced Malignant Disease

**DOI:** 10.3390/cancers13235880

**Published:** 2021-11-23

**Authors:** Mirjam Gerwing, Tobias Krähling, Christoph Schliemann, Saliha Harrach, Christian Schwöppe, Andrew F. Berdel, Sebastian Klein, Wolfgang Hartmann, Eva Wardelmann, Walter L. Heindel, Georg Lenz, Wolfgang E. Berdel, Moritz Wildgruber

**Affiliations:** 1 Clinic of Radiology, University Hospital Muenster, D-48149 Muenster, Germany; tobias.kraehling@ukmuenster.de (T.K.); walter.heindel@ukmuenster.de (W.L.H.); moritz.wildgruber@med.uni-muenchen.de (M.W.); 2 Department of Medicine A, Hematology, Hemostaseology, Oncology and Pulmonology, University Hospital Muenster, D-48149 Muenster, Germany; christoph.schliemann@ukmuenster.de (C.S.); s.harrach@gmx.net (S.H.); christian.schwoeppe@uni-muenster.de (C.S.); andrew.berdel@ukmuenster.de (A.F.B.); georg.lenz@ukmuenster.de (G.L.); berdel@uni-muenster.de (W.E.B.); 3 Gerhard-Domagk-Institute for Pathology, University of Muenster, D-48149 Muenster, Germany; se_klein@t-online.de (S.K.); wolfgang.hartmann@ukmuenster.de (W.H.); eva.wardelmann@ukmuenster.de (E.W.); 4 Department of Radiology, University Hospital, LMU Munich, D-81377 Munich, Germany

**Keywords:** dynamic contrast-enhanced MRI, vascular volume fraction, apparent diffusion coefficient, tTF-NGR, vascular targeting

## Abstract

**Simple Summary:**

Since the knowledge of tumor biology has advanced, a variety of targeted therapies has been developed. These do not immediately affect the tumor size, so optimized oncological imaging is needed. In this phase I study of patients with advanced malignant disease, a multiparametric imaging approach was used to assess changes in tumor perfusion after vessel-occluding therapy with the CD13 targeted truncated tissue factor with a C-terminal NGR-peptide. It comprises different sequences and the use of two different contrast media, ferucarbotran and gadobutrol. This multiparametric MRI protocol enables assessing the therapy effectiveness as early as five hours after therapy initiation.

**Abstract:**

Early assessment of target hit in anti-cancer therapies is a major task in oncologic imaging. In this study, immediate target hit and effectiveness of CD13-targeted tissue factor tTF-NGR in patients with advanced malignant disease enrolled in a phase I trial was assessed using a multiparametric MRI protocol. Seventeen patients with advanced solid malignancies were enrolled in the trial and received tTF-NGR for at least one cycle of five daily infusions. Tumor target lesions were imaged with multiparametric MRI before therapy initiation, five hours after the first infusion and after five days. The imaging protocol comprised ADC, calculated from DWI, and DCE imaging and vascular volume fraction (VVF) assessment. DCE and VVF values decreased within 5 h after therapy initiation, indicating early target hit with a subsequent decrease in tumor perfusion due to selective tumor vessel occlusion and thrombosis induced by tTF-NGR. Simultaneously, ADC values increased at five hours after tTF-NGR administration. In four patients, treatment had to be stopped due to an increase in troponin T hs, with subsequent anticoagulation. In these patients, a reversed effect, with DCE and VVF values increasing and ADC values decreasing, was observed after anticoagulation. Changes in imaging parameters were independent of the mean vessel density determined by immunohistochemistry. By using a multiparametric imaging approach, changes in tumor perfusion after initiation of a tumor vessel occluding therapy can be evaluated as early as five hours after therapy initiation, enabling early assessment of target hit.

## 1. Introduction

The assessment of tumor response to novel targeted agents is one of the major challenges in modern oncological imaging [1]. Measurement of the tumor diameter in CT or MR images using the widely used Response Evaluation Criteria in Solid Tumors (RECIST) criteria [2] is insufficient to assess treatment effectiveness of novel targeted therapies, as their mode of action does not immediately affect the size of the tumor, but targets specific traits [3,4]. For example, pseudoprogression, defined as an initial increase in tumor size due to immune cell infiltrate, edema or/and intratumoral hemorrhage, can mimic progressive disease after treatment with targeted therapies, complicating the adequate evaluation of treatment response [1]. Different variations in RECIST have been introduced so far, inter alia the immune-related RECIST criteria for the response evaluation to immune checkpoint inhibitors [5,6]. These were later revised to a bi-dimensional measurement [7,8] or modified RECIST for the evaluation of hepatocellular carcinomas, which focuses on the viable, contrast-enhancing tumor parts [9]. While these specific criteria aid in differentiating responders from non-responders for some diseases or treatments, not all patients that benefit from a continued therapy can be adequately identified, and a switch to a different therapy can potentially be delayed. While pseudoprogression is typically associated with immunomodulatory therapy, it was also more recently observed after anti-angiogenic treatment with bevacizumab [10]. Therefore, different imaging approaches to evaluate treatment response to novel targeted therapies, including anti-vascular therapy, are needed so that in case of non-response, therapy can be switched early before clinically progressive disease occurs (fail fast).

Targeting the tumor vasculature has been among the first clinically approved attempts of novel anti-cancer drugs, starting with the introduction of the anti-VEGF-A antibody bevacizumab [11]. A wide range of other anti-angiogenic therapies has been introduced since then, as tumors frequently rely on neo-vessel formation, starting from a small diameter of 150–200 µm [12]. Most of these therapies aim to stabilize the disarrayed intratumoral vasculature, with effects already commencing one or two days after treatment initiation [13], but changes in vascular structure and function start at the earliest on day 12; the “normalization window” lasts until approximately day 28 [14]. A new therapy targeting the tumor vasculature, the truncated tissue factor (tTF) with a C-terminal NGR-peptide (tTF-NGR), was recently translated into clinical phase I [15]. tTF-NGR is a pro-coagulatory protein fused to a peptide targeting CD13 preferentially expressed in the tumor vasculature, leading to infarction with subsequent necrosis of the tumor [16,17]. Due to the specific targeting of CD13 and the integrin α_v_β_3_ on the tumor endothelium, no thrombosis is induced in other parenchymatous organs over a therapeutically active dose range. Furthermore, the temporally limited thrombosis enables selective tumor-treatment, and permits other anti-neoplastic therapies to be given in sequence before or after tTF-NGR reaching and accumulating in the tumor, yielding promising combination effects with other antitumor compounds [18].

Imaging the tumor vasculature and the changes under such vascular targeted therapy have been the focus for over a decade, so a wide range of possibilities is available. Besides specific tracers in PET imaging [19,20], methods to assess tumor perfusion, using MRI and CT as surrogate parameters, have been evaluated [21,22]. Derived parameters such as the volume transfer constant (K-trans) additionally provide semi-quantitative measurements of tumor perfusion. While many agents targeting the tumor vasculature exhibit their mode of action within a time frame of days to weeks, tTF-NGR causes a sudden thrombotic occlusion of CD13^+^ tumor vessels within a few hours [15]. Our aim was to assess how different parameters of a multiparametric MRI protocol as early surrogate parameters can detect a target hit and effectiveness concerning the mode of action of tTF-NGR already within a few hours after application of the fusion protein in patients with advanced malignant disease.

We, therefore, analyzed the specific perfusion parameters dynamic contrast-enhanced (DCE) MRI and vascular volume fraction (VVF) and the apparent diffusion coefficient (ADC) in a patient cohort treated with CD13 targeted tTF-NGR.

## 2. Materials and Methods

### 2.1. Patient Characteristics

Data were acquired within the phase I tTF-NGR trial, an open-label, single-arm, non-randomized, prospective single-center study that included patients with recurrent or refractory malignant tumors beyond all standard treatments [15]. The median age of the seventeen patients was 54 years (range 19–74), and four of them were female. Detailed patient characteristics are published (Table S1) [15]. Written informed consent regarding the imaging protocol was obligatory before the first scan and obtained independently from the consent for the phase I study. The phase I study of tTF-NGR was registered accordingly (NCT02902237, EudraCT-No.: 2016-003042-85). The study is presented following the STROBE guidelines.

### 2.2. Treatment

Patients received a daily one-hour infusion of tTF-NGR in 50 mL (later 100 mL) 0.9% NaCl for five consecutive days with a subsequent rest period of two weeks. The starting dose was 1 mg/m^2^/day, with intraindividual dose escalation of 0.5 mg/m^2^, which was changed to 1 mg/m^2^ between 3.0 and 5.0 mg/m^2^, upon the judgment of tolerability. Dose escalation was performed in sequence and not in parallel. In the case of extratumoral thromboembolic events or an increase in Troponin T hs, treatment was stopped, and anticoagulation using enoxaparin (subcutaneous injection of therapeutic doses) and acetylsalicylic acid in different doses was initiated. In this case, MR imaging was performed as early as possible, within the first two days after initiation of anticoagulation [15].

### 2.3. MR Imaging

Baseline MRI was performed before treatment initiation, five hours after the first dose of tTF-NGR and again after five consecutive days of treatment. Imaging was performed on the same scanner, with a standardized protocol for all patients and scans: after an anatomical T2 sequence over the target lesion (breath-triggered, approximately five minutes acquisition time overall), a diffusion-weighted sequence was obtained (4.28 min), followed by the acquisition of T2* maps with an injection of ferucarbotran (5 min) and afterward, DCE imaging with an injection of gadobutrol for 10 min (including pre-scans 12.49 min). Details are provided below. Target lesions were previously identified by means of a CT scan of the chest and abdomen before the first MRI scan. MR imaging was performed with a 1.5 Tesla whole-body scanner (Philips Achieva, Philips Healthcare, Amsterdam, The Netherlands) using a 16-channel sense XL torso coil (Philips Healthcare, Amsterdam, The Netherlands).

For assessment of treatment response, the following parameters were assessed: Vascular volume fraction (VVF) as a measure for the intratumoral blood volume, K-trans as the most established model-based perfusion parameter and apparent diffusion coefficient (ADC) in mm^2^/s as a quantification of the mobility of water molecules that was shown to increase in therapy response [23]. Evaluation of VVF was performed as described previously, adding only a few time points [16]. In brief, T2* maps were acquired during breath-holding every 30 s over a time period of five minutes to assess dynamic changes after manual IV injection of 1.4 mL 0.5 mmol Fe/mL ferucarbotran (Resovist^R^, Nihon Schering, Japan), followed by 20 mL of saline after the acquisition of the first T2* map (first acquired T2* map was native). Afterward, the highest increase in signal intensity in T2* maps was identified. In order to allow for comparison of MRI values between the different scans, normalization on the adjacent autochthonous back muscles was performed; the R2* (1/T2*) values were calculated as *Δ*R2*tumor/*Δ*R2*muscle [24]. The vascular volume fraction characterizes the vascular compartment within the tumor [25]. Its accuracy is not limited by the strong first-pass extravasation of a gadobutrol bolus and the effect of the arterial input function [26,27,28]. This imaging method is a non-invasive surrogate parameter of the perfused tumor blood volume [29].

Dynamic contrast-enhanced MRI was performed after the VVF scan. Injection with a rate of 5 mL/s of gadobutrol in a concentration of 0.1 mmol/kg body weight was started after five dummy scans with an acquisition time of ten minutes. The dummy cycles play out several repetitions of the MRI sequence prior to data collection to allow for system longitudinal and transverse magnetization to reach a steady state. Tumor perfusion was quantified by calculation of K-trans, and a model calculation was used to assess tumoral vasculature [30] using commercially available software (IntelliSpace Portal 9.0, Philips Healthcare, Amsterdam, The Netherlands). This technique is based on modeling concentration changes in the contrast agent with pharmacokinetic methods. The signal intensity is converted into a concentration, and concentration versus time curves are fitted using a bicompartmental model comprised of vessels and extravascular extracellular space [31]. The pharmacokinetic is performed on a pixel-by-pixel basis based on the Extended Tofts Model [32]:

*C(t)* = *v*_*p*_
*C*_*a*_
*(t)* + *K*^*trans*^
*e*^−*tk*^_*ep*_ ∗ *C*_*a*_
*(t)*

where *C(t)* is the contrast concentration in tissue, *v_p_* is the fractional value of blood plasma, *C_a_ (t)* is the arterial input function (contrast concentration in feeding artery), *K^trans^* is the transfer constant between blood plasma and extravascular extracellular space (EES) and *k_ep_* is the rate constant between EES and blood plasma. For the calculation of permeability characteristics, a model-based AIF was used, based on the use of Gd-DTPA, an injection dose of 0.1 mmol/kg, a medium injection duration of 5–10 s and a normal hematocrit of 45% (see handbook IntelliSpace Portal 9.0). K-trans is affected by vascular permeability, blood perfusion and vascular surface area [33], but utility and interpretation can be a challenge [30].

Apparent diffusion coefficient (ADC) was calculated from diffusion-weighted imaging (b-values 0, 300 and 600). Regions Of Interest (ROIs) were placed in the target lesions at a comparable position in the pre-and post-therapeutic scans, as well as in follow-up scans.

The rationale of this study was to compare the different methods of non-invasive MRI measurement as early surrogate parameters to detect early target hit and tumor vessel occlusion effectiveness with reference to the mode of action of tTF-NGR, to identify the most suitable one and evaluate differences between these methods due to their specific approach.

### 2.4. Immunohistochemistry (IHC) Staining

In order to assess vascular density in individual tumors, three different stainings were used: platelet endothelial cell adhesion molecule (CD31), hematopoietic progenitor-cell antigen (CD34) and early growth response gene (EGR). The mean vessel density per µm was defined as the mean value of all three different stainings. The following primary antibodies were applied: CD34 (QBEndD, Roche Diagnostics, Rotkreuz, Switzerland, prediluted), CD31 (JC70, Cell Marque, Rocklin, CA, USA, prediluted) and ERG (EPR38/64, Roche Diagnostics, Rotkreuz, Switzerland). IHC staining was performed with a Benchmark ULTRA Autostainer (VENTANA/Roche Diagnostics, Rotkreuz, Switzerland) on 3 µm sections. In brief, the staining procedure included heat-induced epitope retrieval pretreatment using Tris-Borate-EDTA buffer (pH 7.8; 95–100 °C, 32–72 min) followed by incubation with respective primary antibodies for 16 to 120 min and employment of the OptiView DAB IHC Detection Kit (VENTANA/Roche Diagnostics, Rotkreuz, Switzerland).

Respective slides for CD31, ERG and CD34 stainings were digitized using a Leica SCN400 slide scanner (Leica Biosystems, Wetzlar, Germany) at 40× magnification. Virtual-whole slide images were processed as described previously [34], the microvessel density (MVD) was calculated using a clustering algorithm for the given vascular markers [35]. Biopsy samples were obtained prior to screening for this study.

### 2.5. Statistical Analysis

A comparison of the three time points was performed using a one-way ANOVA, and adjusted *p*-values were calculated with Tukey’s multiple comparison. The correlation between parameters was assessed using a two-tailed Spearman test. The *p*-values below 0.05 were considered significant. Statistical analysis was performed using GraphPadPrism 9 (GraphPad Software Inc., La Jolla, CA, USA).

## 3. Results

All patients tolerated the injection of contrast media with a short time interval in between well and did not exhibit any side effects.

### 3.1. Imaging of Target Hit and Effectiveness

**Figure 1:**
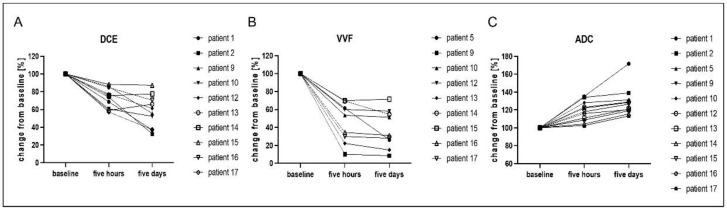
Multiparametric MR imaging shows a decrease in perfusion after therapy with tTF-NGR. Overview of the change in perfusion parameters DCE (measured as k-trans) (**A**) and vascular volume fraction (VVF) (**B**) in comparison to the ADC value (**C**) over the course of the first therapy cycle with tTF-NGR. MRI assessment was performed before therapy initiation, five hours after the first infusion and after five days of therapy. A decrease in perfusion parameters DCE and VVF and an increase in ADC were assessable as early as five hours after the first therapy, with an even further reduction after five days of therapy in some of the patients. Values are given as the change from baseline (set to 100% for each individual patient) for better comparison of the three evaluated parameters. One-way ANOVA revealed high statistical significance of *p* < 0.0001 overall for all three parameters. Not all imaging techniques for all time points were available (see manuscript for details), so only data of patients with completed imaging and analysis are shown here.

For comparison of the different imaging approaches, values are presented as percentage changes from the baseline scan prior to the first tTF-NGR dose application (Figure 1).

**Figure 2:**
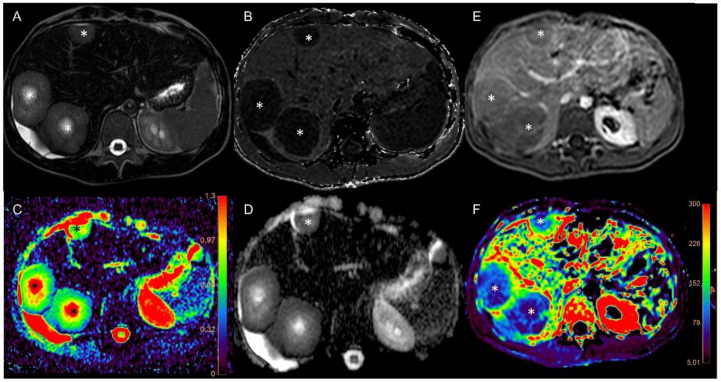
Exemplary imaging data. Imaging of patient 13 with a desmoplastic round cell tumor with multiple liver metastases (marked with an asterisk) before therapy. (**A**) T2 for anatomical information, (**B**) T2* after injection of ferucarbotran for calculation of VVF, (**C**) ADC map, (**D**) ADC, (**E**) DCE scan after injection of gadobutrol, (**F**) calculated K-trans map. The imaging protocol thus comprises different sequences to first acquire anatomical information, followed by different possibilities for the assessment of tumor perfusion and its changes after therapy. Color scale in C represents the ADC value, color scale in F represents the k-trans value.

**Figure 3:**
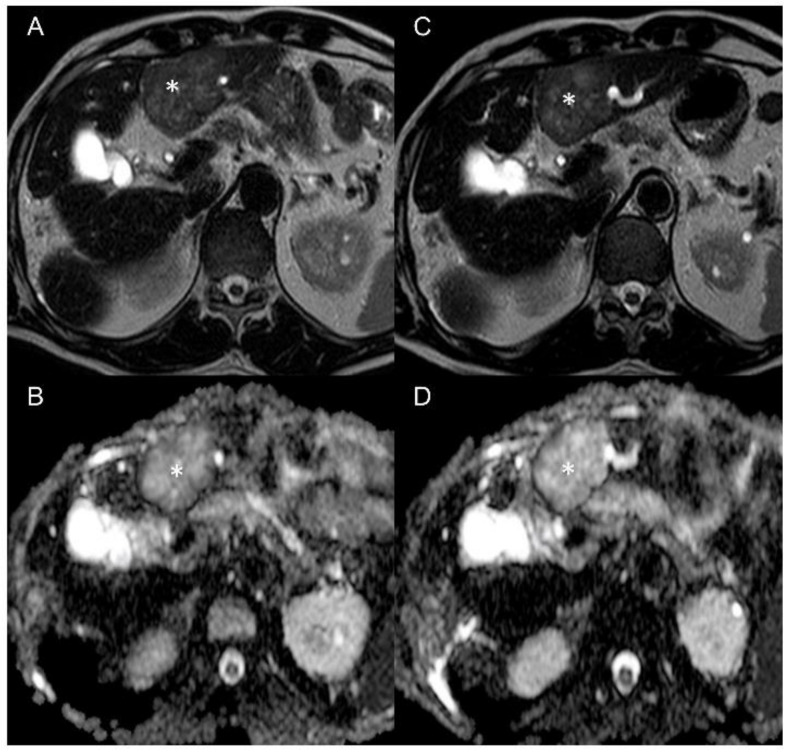
Imaging of patient 16 with colorectal carcinoma and liver metastases (marked with an asterisk) before (**A**,**B**) and after (**C**,**D**) the second cycle shows an increase in ADC value (**B** + **D**) and changes in T2 weighted imaging (**A** + **C**). These morphological changes can be further assessed with the multiparametric MRI protocol.

Due to the poor condition of patients with advanced malignancies, the imaging protocol had to be shortened in some cases, so not all imaging data were available for every time point in each patient. All available data were included in the analysis. DCE and VVF values dropped significantly in all patients five hours after the first infusion of tTF-NGR in comparison to the pretherapeutic scan and decreased further after five consecutive days of treatment (one-way ANOVA *p* < 0.0001 for both parameters overall). DCE values decreased to 73 ± 11% in the five-hour scans (adjusted *p*-value 0.0001) and further to 58 ± 18 % (adjusted *p*-value 0.0273 compared to five-hours scan and *p* < 0.0001 compared to baseline) after five consecutive days of treatment. VVF values decreased to 57 ± 22 % (adjusted *p*-value < 0.0001) five hours after treatment initiation and to 38 ± 21% (adjusted *p*-value 0.6550 compared to five-hours scan and adjusted *p*-value < 0.0001 compared to baseline) after five days of tTF-NGR treatment. An example of the acquired imaging data is shown in Figure 2. In contrast, ADC values were elevated after the first scan to 119 ± 11 % (adjusted *p*-value 0.0018) with a further increase after five days of therapy to 129 ± 16 % (adjusted *p*-value 0.1066 compared to five-hour scan and adjusted *p*-value < 0.0001 compared to baseline). The one-way ANOVA for ADC values showed *p* < 0.0001 (Figure 3).

### 3.2. Correlation Analysis

**Figure 4:**
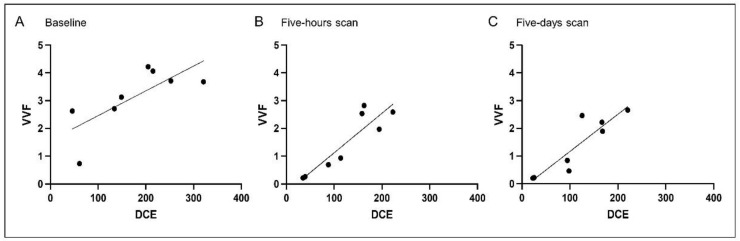
Correlation between imaging parameters DCE and VVF before and after therapy with tTF-NGR. Correlation of the values of DCE and VVF over the first cycle of tTF-NGR therapy visualizes that either value can be used for assessment of perfusion after treatment targeting the tumor vasculature, for the pre-scan (**A**), as well as for the scan after five hours (**B**) and five days (**C**). Spearman two-tailed correlation revealed a significant correlation between the respecting values over the entire course of the first cycle (baseline scan r = 0.046, *p* = 0.046, five-hours scan r = 0.881, *p* = 0.007, five-days scan r = 0.881, *p* = 0.007).

**Figure 5:**
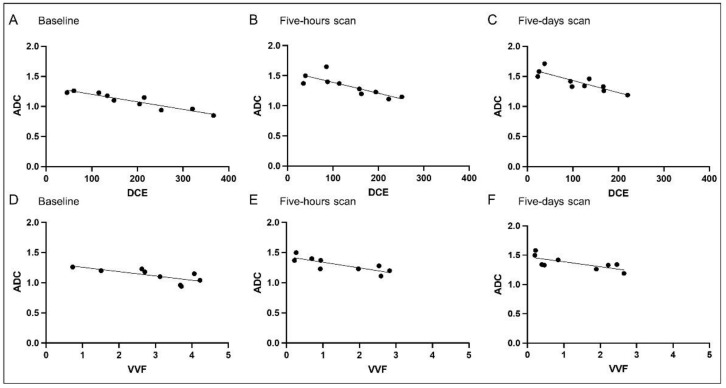
Correlation between DCE and ADC as well as VVF and ADC imaging parameters before and after therapy with tTF-NGR. Correlation of DCE and ADC (**A**–**C**) as well as VVF and ADC (**D**–**F**) shows a good correlation of the individual perfusion parameters with the ADC values. Spearman two-tailed correlation revealed a significant correlation between the respecting values over the course of the first cycle (**A**–**C** baseline scan r = −0.9301, *p* = 0.0003, five-hours scan r = 0.8693, *p* = 0.0018, five-days scan r = −0.8511, *p* = 0.0029; **D**–**F** baseline scan r = −0.8, *p* = 0.0138, five-hours scan r = −0.8068, *p* = 0.0119, five-days scan r = −0.7395, *p* = 9.0275).

In order to evaluate if the parameters of the different imaging approaches that rely on different models and assumptions correlate, a correlation analysis was performed. VVF and DCE showed a moderate correlation for the baseline scan (r = 0.046, *p* = 0.046), and a good correlation for the post-therapeutic scans (five-hours scan r = 0.881, *p* = 0.007, five-days scan r = 0.881, *p* = 0.007) (Figure 4). Reverse correlation of DCE and ADC values were also significant (baseline scan r = −0.9301, *p* = 0.0003, five-hours scan r = 0.8693, *p* = 0.0018, five-days scan r = −0.8511, *p* = 0.0029), as were the reverse correlation coefficients for VVF and ADC (baseline scan r = −0.8, *p* = 0.0138, five-hours scan r = −0.8068, *p* = 0.0119, five-days scan r = −0.7395, *p* = 9.0275) (Figure 5). Nine of the patients received a second cycle, most of those showing a recovery of the tumor perfusion in the two weeks between the end of the first cycle and the baseline scan for the second cycle (Figure S1).

### 3.3. Evaluation of Reversed Effects Due to Therapeutic Anticoagulation

**Figure 6:**
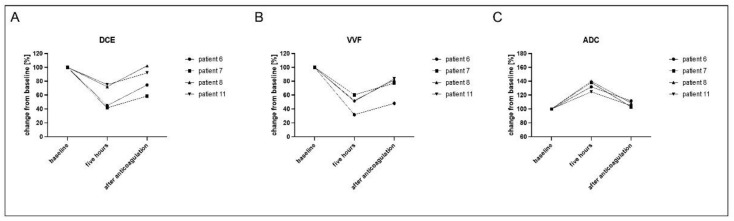
All imaging parameters show a fast normalization of perfusion after effective anticoagulation. Four patients needed a withdrawal of the study medication tTF-NGR due to elevation of troponin T hs or Grade 2 thrombotic events. These patients were immediately anticoagulated with enoxaparin, patient 6 and patient 11 on day 3, patient 7 on day 1 and patient 8 on day 2. Imaging was performed one day after anticoagulation (patient 6, patient 8) or two days after anticoagulation (patient 7, patient 11) and showed the reversibility of the therapeutic effect of tTF-NGR on the parameters DCE (**A**), VVF (**B**) and ADC (**C**). Overall one-way ANOVA was significant for all three parameters (DCE *p* = 0.0209, VVF *p* = 0.0044, ADC *p* = 0.0010).

Four patients were discontinued on study medication due to adverse side effects, and effective anticoagulation was initiated (Figure 6). These patients were imaged within two days after anticoagulation to assess the dynamics of the anticoagulatory effect and evaluate if the pro-thrombotic effect of tTF-NGR persists shortly after induction of anticoagulation.

VVF, DCE and ADC values of these patients exhibited effective anticoagulation with reverse effects, with values of DCE and VVF increasing again (DCE 58 ± 18 in the five-hour scan, 82 ± 19 after anticoagulation, adjusted *p*-values 0.0361 baseline vs. five hours, 0.0165 five hours vs. after anticoagulation, 0.2862 baseline vs. after anticoagulation; VVF 49 ± 12 in the five-hours scan, 73 ± 17 after anticoagulation, adjusted *p*-values 0.0071 baseline vs. five hours, 0.0220 five hours vs. after anticoagulation, 0.0906 baseline vs. after anticoagulation), and ADC values decreasing (134 ± 7 in the five-hours scan, 107 ± 4 after anticoagulation, adjusted *p*-values 0.0047 baseline vs. five hours scan, 0.0128 five hours vs. after anticoagulation, 0.0756 baseline vs. after anticoagulation).

### 3.4. Correlation with Immunohistochemistry (IHC)

The correlation of the mean vessel density as determined by immunohistochemical staining and changes in imaging parameters did not correlate significantly (Table 1). This indicates that the vascular occlusion effectiveness of tTF-NGR leading to a decrease in tumor perfusion is not dependent on the tumoral vascular density within the tumor lesions, and tumor vessel density is not a good selection parameter for patients before treatment with tTF-NGR.

## 4. Discussion

In this study, a multiparametric MR imaging approach was used to assess the immediate effects of a novel CD13 targeted therapy with tTF-NGR on the tumor vasculature and the resulting changes in tumor perfusion. This therapy leads to intratumoral thrombotic vascular occlusion, a significant reduction in tumor perfusion, and subsequent tumor infarction leading to necrosis in preclinical models [16]. The assessment of the baseline tumor perfusion and subsequent changes under treatment is possible with a variety of different imaging approaches [1], which, however, often face the challenge of varying protocols among institutions and non-standardized options for postprocessing analysis [36].

DCE-MRI has been widely used for diagnosis and staging of cancer, as well as tumor response assessment [37,38]. It has already been applied as a secondary endpoint in several studies designed to evaluate responses to anti-angiogenic agents and other agents targeting the tumor vasculature [38]. However, it lacks high accuracy and is limited by strong first-pass extravasation of the contrast agent bolus and by effects of the arterial input function [26,27,28]. The calculation of the perfusion parameters based on an arterial input function might also be adversely influenced by partial volume effects, signal nonlinearity, B1 inhomogeneity and inadequate temporal resolution [39]. For anti-vascular therapies that do not target VEGF and have a different mode of action, changes in the microvasculature independent of changes in the vascular permeability might not be sufficiently assessed using DCE-MRI, as the derived parameters such as K-trans may remain unchanged in the case of smaller microvascular changes or a decrease in vessel density [24]. DCE, with the use of gadofosveset, an albumin-binding gadolinium-based contrast agent, has already proven to visualize early treatment response in mice treated with tTF-NGR [40]. As a different approach, measurement of the R* with ferucarbotran is insensitive to large-scale field inhomogeneities and thus a robust tool for quantitative monitoring of tumor vascularization for treatment monitoring [29]. It is scanned during breath-holding, so breathing artifacts can largely be avoided in comparison to the continuous measurement in DCE imaging. It also avoids the trade-off between high speed and limited resolution with DCE-MRI [24]. In the first in-man-experience of tTF-NGR, this approach was able to show reduced tumor perfusion after the first therapy in well-vascularized lesions in some patients [16], which was confirmed in the phase I study with much higher doses of tTF-NGR reached. A similar approach, using a different iron-based contrast medium (SHU 555 C), was used in a mouse study with tTF-NGR, which also enabled to visualize a reduced tumor perfusion after thrombotic occlusion of tumor vessels [22]. However, superparamagnetic iron oxide particles are not used in clinical routine anymore, so currently no standardized protocol exists.

Using a combination of DCE and VVF imaging enables not only assessing changes in permeability and perfusion but also smaller changes in the microvascular compartment.

ADC measures the movement of water molecules and can be used for different implications in oncologic imaging, such as differentiation of malignant from non-malignant lesions and tumor heterogeneity [41]. It has been used in clinical studies before the widespread use of targeted therapies [42] and is increasingly used to assess therapy-induced changes after anti-angiogenic therapy [43,44]. After initiation of chemotherapy treatment, diffusion imaging can predict tumor response as early as 4 or 11 days after commencement of therapy [42]. An increase in ADC values over the duration of various anti-cancer treatments was since correlated with therapy response in different solid tumors, e.g., liver metastases of breast cancer [42], rhabdomyosarcoma [45], colorectal liver metastases [46] and cholangiocarcinoma [47]. Interestingly, we could observe an early increase in ADC values upon application of tTF-NGR. In patients with well-circumscribed HCC, an increase of 35 % in ADC values after TACE correlated with a particularly good outcome after therapy; in our cohort, a mean increase of 28.7% was achieved [48].

In order to enable an accurate measurement of changes in tumor perfusion and vascular structure after treatment with tTF-NGR shortly after therapy initiation, these different imaging approaches were combined. The half-life of ferucarbotran is divided into two phases, with the first clearance of about 80% after 3.9–5.8 min and the second phase of 2.4–3.6 h [49]. In a study evaluating the blood signal, it was at 12% 15 min after administration; another 15 min later, it was below noise level [50]. Therefore, in this study protocol, a great part of ferucarbotran was cleared before the injection of gadobutrol. The half-life of gadolinium agents is 1.5 h (when renal function is normal) longer; after 12 h, > 90% recovered [51], so it was injected as the second contrast agent.

In the assessment of tumor therapy, the assessment of initial changes is important to allow for a timely treatment change in case of failed treatment response. This study revealed that all three approaches provide complementary information on the tumor vasculature as early as 5 h after the first infusion of tumor vessel occluding tTF-NGR, thus serving as an early surrogate parameter to detect a target hit and tumor vessel occlusion effectiveness with reference to the mode of action of tTF-NGR. Furthermore, the prompt recovery of perfusion upon anticoagulation was measurable, which highlights the specificity of the observations and hints towards options for safety guidance of tTF-NGR.

Determination of mean vessel density in IHC did not correlate with response in imaging parameters. This indicates that the vascular occlusion effectiveness of tTF-NGR leading to a decrease in tumor perfusion is not dependent on the pre-existing vascular density within the tumor lesions. This underlines the rationale of testing tTF-NGR in further studies independent of the degree of vascularity of the tumor but to concentrate on target positivity on the tumor vessels or tumor cells instead.

The most important limitation of this study is the low number of patients in this phase I study, with missing imaging data for certain time points, as not all patients were able to undergo the entire imaging protocol. Furthermore, ferucarbotran and gadobutrol were injected in one MRI measurement, so ferucarbotran was not entirely cleared before the injection of gadobutrol. While we assessed the effects of ferucarbotran with T2* maps and the effects of gadobutrol with T1 weighted DCE images, the latter absolute values might have been influenced by the remaining ferucarbotran. Therefore, we used a standardized protocol for all measurements so that the changes in comparison to baseline were all influenced in a similar way. This protocol was chosen to avoid the stress of additional MRI scans in patients with advanced malignant disease. Due to the low number of patients and the dose escalation protocol for tTF-NGR, we did not attempt to correlate imaging effects to dose level but concentrated on the global effect sizes observed.

## 5. Conclusions

This multiparametric imaging approach allowed for visualization of changes in tumor perfusion already five hours after treatment initiation with anti-vascular tumor therapy.

The rapid assessment on the day of treatment initiation can serve as an early surrogate parameter to detect a target hit and tumor vessel occlusion effectiveness with reference to the mode of action of tTF-NGR and, possibly, also other anti-vascular therapies.

## Figures and Tables

**Table 1:** Correlation between imaging parameters and mean vessel density. Neither DCE, VVF nor ADC correlate with the mean vessel density per µm, as determined using immunohistochemical staining of the patients’ slices with ERG, CD31 and CD34. Therefore, assessment of the mean vessel intensity is not suitable for patient selection before therapy with tTF-NGR.

Parameter	Baseline	Five-Hours Scan	Five-Days Scan
DCE	r = 0.1084 *p* = 0.838	r = 0.1539 *p* = 0.771	r = 0.08016 *p* = 0.88
VVF	r = −0.5441 *p* = 0.4559	r = −0.1657 *p* = 0.8343	r = −0.2757 *p* = 0.7243
ADC	r = −0.1724 *p* = 0.7439	r = −0.2192 *p* = 0.6765	r = −0.3883 *p* = 0.5184

## Data Availability

The data presented in this study are available on request from the corresponding author. Further data of this study has been published previously [15].

## Supplementary Materials

The following are available online at https://www.mdpi.com/article/10.3390/cancers13235880/s1, Figure S1: Recovery of all imaging parameters after the first cycle. Overview over the different values visualizes a recovery of all three parameters between cycle one and cycle two in the therapy free interval of two weeks. It further shows a repeated decrease in perfusion parameters k-trans (**A**) and VVF (**B**) and an increase in ADC (**C**) for the second cycle, Table S1: Overview over patient characteristics.
