# Supplementary material for: Multiparametric Magnetic Resonance Imaging for Immediate Target Hit Assessment of CD13—Targeted Tissue Factor tTF-NGR in Advanced Malignant Disease
Source: Cancers (Basel). 2021 Nov 23;13(23):5880. doi: 10.3390/cancers13235880 (PMC8657298; doi:10.3390/cancers13235880)
Supplement: Supplementary file 1 [file cancers-13-05880-s001.zip › cancers-1432721-supplementary.pdf]

Supplementary Materials: Multiparametric Magnetic Resonance Imaging for Immediate Target Hit Assessment of CD13 - Targeted Tissue Factor tTF-NGR in Advanced Malignant Disease

Mirjam Gerwing, Tobias Krähling, Christoph Schliemann, Saliha Harrach, Christian Schwöppe, Andrew F. Berdel, Sebastian Klein, Wolfgang Hartmann, Eva Wardelmann, Walter Heindel, Georg Lenz, Wolfgang E. Berdel and Moritz Wildgruber

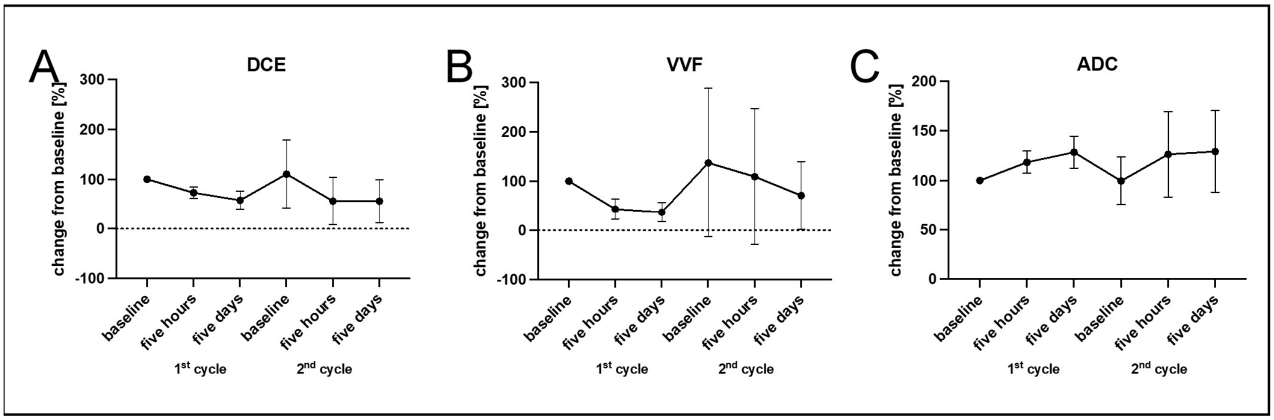

Figure S1. Recovery of all imaging parameters after the first cycle. Overview over the different values visualizes a recovery of all three parameters between cycle one and cycle two in the therapy free interval of two weeks. It further shows a repeated decrease in perfusion parameters k-trans (A) and VVF (B) and an increase in ADC (C) for the second cycle.

Table S1. Overview over patient characteristics PEI; Cisplatin, Etoposide, Ifosfamide (Ifo); HD-PEI, high-dose PEI with stem cell rescue; TI, Paclitaxel, Ifo; TIP, TI plus Cisplatin; HD-CE, HD Carboplatin, Etoposide; Gem/Ox, Gemcitabine, Oxaliplatin; TACE, transarterial chemoembolization; CNS-PCI, prophylactical brain irradiation; ACO, Doxorubicin, Cyclophosphamide, Vincristine; HIPEC, hyperthermic intraperitoneal chemotherapy; FOLFOX, 5-Fluorouracil, folinic acid, Oxaliplatin; Bev, Bevacizumab; FOLFIRI, 5-Fluorouracil, folinic acid, Irinotecan; SIRT, selective internal radiotherapy; Doxo/Ifo, Doxorubicin, Ifosfamide; Gem/Doc, Gemcitabine, Docetaxel; VIDE, Vincristine, Ifo, Doxo, Etoposide; VAI, Vincristine, Actinomycin, Ifo; FUO, fever of unknown origin; DVT, deep vein thrombosis; TIA, transient ischemic attack; DLT, dose limiting toxicity; IMP, investigational medicinal product; RECIST PD, progressive according to RECIST criteria.

Patient Number	Gender (m/f)	Age [Y]	Diagnosis	Previous Therapy	tTF-NGR cycles	tTF-NGR Dose [mg/m^2]	DLT related to IMP	Comments
1	m	22	metastatic non-seminomatous germ cell tumor	PEI, HD-PEI, surgery, TI, TIP, HD-CE, Gem/Ox, Radiation, Nivolumab	2	cycle 1: 1.0<br>cycle 2: 1.5	good tolerability, no DLT	RECIST PD
2	f	29	hepatocellular carcinoma + malignant ascites, metastatic	surgery, Sorafenib, Regorafenib, TACE	2	cycle 1: 1.5<br>cycle 2: 2.0	good tolerability, no DLT	RECIST PD
3	m	63	small cell lung (SCLC), extensive	radiochemo CE, CNS-PCI, CE, Topotecan, ACO	2	cycle 1: 2.0<br>cycle 2: 2.5	good tolerability, no DLT	RECIST PD

			disease, metastatic					
4	m	46	rectal adeno, metastatic	neoadj. radiochemo, surgery + HIPEC, FOLFOX, liver surgery, radiation, FOLFIRI + Bev, SIRT, FOLFOX + Bev, TAS-102 (Trifluridin/Tipiracil)	2	cycle 1: 2.5<br>cycle 2: 3.0	good tolerability, no DLT	RECIST PD
5	m	58	colon adeno, metastatic RAS-mutated	surgery, FOLFOX + Bev, FOLFIRI + Bev, Ramucirumab, TAS-102	3	cycle 1: 3.0<br>cycle 2: 4.0<br>cycle 3: 5.0	good tolerability, no DLT	RECIST PD
6	m	45	synovial sarcoma, metastatic	surgery, Doxo/Ifo, Trabectedin, Gem/Doc, Doxo + Fibromun (study), Pazopanib, Bendamustin, Vandetanib, HD-Ifo	1	Cycle 1: 5.0 (3 days)	DLT, related to IMP, Troponin T hs, grade 3;<br>DVT, related to IMP, grade 2	DLT (Trop) and DVT reversible<br>RECIST PD
7	m	56	lung squamous, metastatic	radiochemo, Cisplatin/Vinorelbin, Nivolumab, Docetaxel, Gemcitabine, Afatinib	1	cycle 1: 5.0 (1 day)	DLT, related to IMP, Troponin T hs, grade 3	DLT (Trop) reversible<br>RECIST PD
8	m	74	colon adeno, metastatic	surgery, FOLFOX + Bev, FOLF + Bev + anti-PDL-1 (study), FOLFIRI + Cetuximab, FOLFOX + Cetuximab	1	cycle 1: 4.0 (2 days)	DLT, related to IMP, Troponin T hs, grade 3	DLT (Trop) reversible<br>RECIST PD
9	m	26	extragonadal germ cell tumor, metastatic	PEI, surgery, TI, TIP, HD-CE, radiation, Gem/Ox/Paclitaxel	1	cycle 1: 4.0	good tolerability, no DLT	only 1 cycle since fast RECIST PD
10	m	63	medullary thyroid cancer	surgery, Vandetanib, Cabozantinib	1 (completed)	cycle 1: 4.0<br>cycle 2: 4.0 (1 day)	cycle 1: good tolerability, no DLT; cycle 2: catheter-associated DVT related to IMP, grade 2	catheter-associated DVT reversible<br>RECIST PD
11	f	54	undifferentiated teratoma, then squamous carcinoma, CUP (ovary?), metastatic	surgery, PEB, PEI, Carboplatin/Paclitaxel	1	cycle 1: 4.0 (3 days)	DLT, related to IMP, Troponin T hs, grade 3	DLT (Trop) reversible<br>RECIST PD
12	f	41	angiosarcoma of the heart, metastatic	surgery, radiochemo, Paclitaxel, Doxo/Ifo, Pazopanib, radiation, Gem/Doc, Gem, Trabectedin, SIRT	1	cycle 1: 3.0<br>cycle 2: 3.0 (1 day)	cycle 1: good tolerability; cycle 2: TIA, related to IMP, grade 2	TIA reversible<br>RECIST PD
13	m	19	desmoplastic small/round cell tumor, metastatic	VIDE, VAI, Temodal/Irinitecan	2	cycle 1: 3.0<br>cycle 2: 3.0	good tolerability, no DLT	RECIST PD

14	m	63	synovial sarcoma, metastatic	surgery, radiation, Doxo/Ifo, Trabectedin, Gem/Doc	1	cycle 1: 3.0 (1 day)	DLT, related to IMP, Troponin T hs, grade 3	DLT (Trop) reversible RECIST PD
15	f	56	hepatocellular carcinoma, metastatic	TACE, SIRT, Sorafenib (toxicity), Nivolumab (toxicity)	2	cycle 1: 3.0 cycle 2: 3.0	good tolerability	RECIST PD
16	m	60	colon adeno, metastatic	FOLFIRI-OX + Panitumumab, 5-FU + Panitumumab, radiotherapy, FOLFIRI + Bevacizumab, Trifluridin/Tipiracil, Irinotecan + Cetuximab	2	cycle 1: 3.0 cycle 2: 3.0	good tolerability	RECIST PD
17	m	24	undifferentiated pleomorphic sarcoma, metastatic	EURAMOS protocol (Cisplatin, Doxo, HD-MTX and surgery), adjuvant Mifamurtid, Ifo + Etoposide, surgery of metastasis, radiotherapy, Gem/Doc, Nivolumab, Pazopanib	2	cycle 1: 3.0 cycle 2: 3.0	good tolerability	RECIST PD

Table S1 was modified from Table 1 in the original publication of the phase I study [1].

Reference

1. Schliemann, C.; Gerwing, M.; Heinzow, H.; Harrach, S.; Schwöppe, C.; Wildgruber, M.; Hansmeier, A.A.; Angenendt, L.; Berdel, A.F.; Stalmann, U.; et al. First-In-Class CD13-Targeted Tissue Factor tTF-NGR in Patients with Recurrent or Refractory Malignant Tumors: Results of a Phase I Dose-Escalation Study. Cancers (Basel) 2020, 12, doi:10.3390/cancers12061488.
